# Gastrointestinal Kaposi: A Rare Case Unveiling the Presentation and Management Challenges of an Uncommon Neoplasm in the Digestive Tract

**DOI:** 10.7759/cureus.56892

**Published:** 2024-03-25

**Authors:** Santiago Philibert-Rosas, Ruth Rabago Escoto, Ariosto H Hernandez Lara, Ceriolith Tenorio Flores, Edwin Ornelas Escobedo

**Affiliations:** 1 Department of Health Sciences, Universidad Anahuac Mexico, Mexico City, MEX; 2 Gastrointestinal Endoscopy, Mexico's General Hospital, Mexico City, MEX; 3 Research-Innovation Department, Institut Hospitalo-Universitaire de Strasbourg, Strasbourg, FRA

**Keywords:** sexually transmitted diseses, herpes virus type 8( hhv-8), upper endoscopy, hiv aids, gastrointestinal kaposi's sarcoma

## Abstract

Gastrointestinal Kaposi’s sarcoma (GI-KS), which is frequently observed in individuals with HIV/AIDS, tends to manifest with vague symptoms or may not show any symptoms at all. These symptoms can include abdominal discomfort, nausea, vomiting, and low levels of iron in the blood, and they may worsen as the tumor enlarges, leading to more severe issues such as blockage or perforation of the bowel. Diagnosis usually requires an endoscopy to confirm the presence of GI-SK in individuals showing symptoms. In this case report, we describe a 29-year-old Hispanic male with vague symptomatology, anemia, and a probable unknown bleeding site.

## Introduction

Kaposi sarcoma (KS), initially identified in 1872 by Moritz K. Kaposi [1], is an angioproliferative tumor caused by the human herpesvirus 8 (HHV-8). KS manifests in four distinct variants: (1) classic KS, typically affecting the lower extremities and infrequently involving visceral organs, (2) endemic KS, prevalent in Africa and primarily affecting young males, with incidence rising with age, (3) immunosuppression-related KS, occurring in post-transplant or immunosuppressive therapy recipients, (4) AIDS-related KS, the most aggressive form, is characterized by skin lesions and mucosal or visceral involvement in 20-25% and 15% of cases, respectively [2,3].

Gastrointestinal Kaposi’s sarcoma (GI-KS) lesions, which are associated with HIV infection, can manifest independently of any visible signs on the skin. While some individuals may experience no symptoms, others might experience abdominal pain, weight loss, nausea, vomiting, malabsorption, or diarrhea [4]. However, as the disease progresses, these lesions can cause more severe and life-threatening complications such as gastrointestinal bleeding, persistent abdominal pain, gastric outlet obstruction, or even intussusception, a condition where one segment of the intestine invaginates into another segment. These symptoms can significantly impact the quality of life and may require prompt medical intervention for proper management [5,6].

## Case presentation

**Figure 1:**
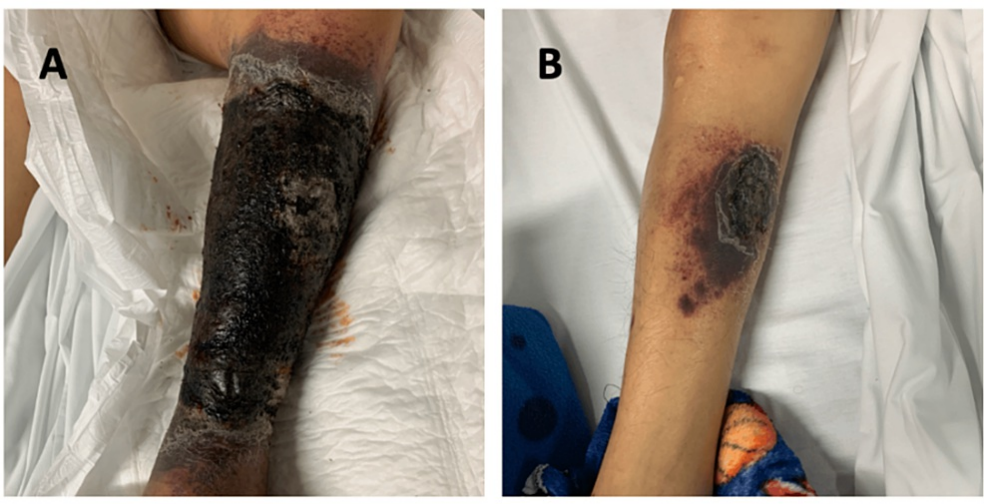
A. Left leg lesion covering the whole tibial area. B. Right leg lesion.

**Figure 2:**
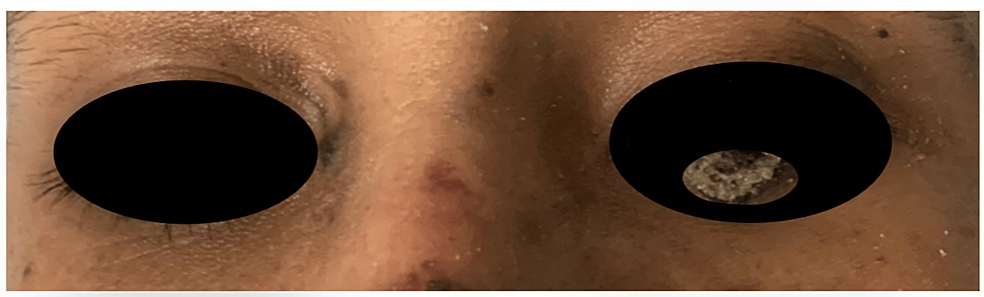
Lower left eyelid lesion.

A 29-year-old Hispanic male first noticed a 3cm x 3cm hematoma in the tibial region of the left leg, which disappeared. A month later, it reappeared in a larger size, measuring 7cm x 5cm, painless with changes in coloration, tenderness to palpation, and increased size (Figure 1A). He consulted an external physician who initiated management with homeopathic medications, dexamethasone, and vitamin K. Subsequently, he reported that the lesion continued to grow in size until it covered the whole tibial area, causing scarring and occasional bleeding. Following the worsening lesion, a week later, the patient began to notice a hematoma in the tibial area of the right leg, approximately 2 cm x 5cm, with changes in coloration, edema, and throbbing pain (Figure 1B). Days later, he noticed multiple lesions in the facial region, specifically on the lower left eyelid, chest, and arms, which were purplish, non-painful, non-edematous, and non-hemorrhagic (Figure 2). In addition to the presenting symptoms and sexual history, it is essential to note that the patient initiated sexual relations at the age of 17 without contraceptive barriers and has had multiple sexual partners. In these instances, male-to-male intercourse was mentioned but not confirmed by the patient. He underscored that he has undergone two HIV rapid tests, yielding negative results on both occasions. 

Prompted by these developments, the patient sought medical attention at the emergency department. Upon arrival, the patient presented with blood pressure (BP) of 90/60 mmHg, mean arterial pressure (MAP) of 70 mmHg, heart rate (HR) of 120 beats per minute, and respiration rate (RR) of 22 per minute. Lab tests were ordered, revealing metabolic acidosis with hyperlactemia, hyponatremia with hypoosmolarity, hypocalcemia, hypoalbuminemia, normocytic normochromic anemia (13 g/dL), and moderate thrombocytopenia (52,000). In response to diagnostic findings, management was provided with crystalloid solutions, buprenorphine-based analgesia, proton pump inhibitors, dual antimicrobial therapy with an unknown cephalosporin, and clindamycin. A rheumatology and infectious disease consultation was requested at the same institution where he arrived. 

During the next 36 hours in the emergency department, the patient experienced worsening of the lesions in the pelvic limbs, with the lesion exhibiting progressive enlargement without any signs of bleeding or loss or impaired function, measuring 30cm x 15cm on the left leg and 9cm x 4cm on the right leg. However, both lesions showed suggestive signs of necrosis. Lab controls report decreased hemoglobin (12.2 g/dL) and platelets (39,000). The infectious disease department decided to admit the patient due to suspicion of HIV infection. 

A week after admission, the patient was referred to the endoscopy department with a diagnosis of HIV and normocytic normochromic anemia type III with hemoglobin of 6.3g/dl. The patient also presented with moderate thrombocytopenia (82,000 platelets) and leukopenia (4.2 white blood cells). Hemoglobin levels were subsequently improved to 7.2g/dl.

The referral was prompted by concerns of upper gastrointestinal bleeding to account for the rapid decrease in hemoglobin. However, it is essential to note that the patient did not exhibit any symptoms suggestive of upper or lower gastrointestinal bleeding, such as coffee ground emesis, melena, rectal bleeding, or hematochezia. 

The patient was stable and cooperative, with multiple purplish lesions covering from face to feet and involving arms and legs. Notably, lesions in the tibial area of both legs had been visible since the patient's arrival at the emergency department. Further investigation revealed a Kaposi immunophenotype positive for Ki67, CD31, and HHV-8, confirming a suspected KS diagnosis. This confirmation was obtained through a biopsy of the patient’s left arm skin. 

The endoscopic procedure was initiated with the patient’s prior consent, and local anesthesia was administered orally using lidocaine. For this study, an upper endoscopy procedure was performed by experienced specialists utilizing an advanced endoscope, model EG-29i10, manufactured by Pentax (Tokyo, Japan). The patient was positioned comfortably on their left side during the upper endoscopy. After administering local anesthesia to the throat, the endoscope was carefully inserted through the oral cavity and advanced gently into the esophagus, stomach, and duodenum. The endoscope’s high-definition camera provided clear, detailed images of the mucosal lining and structures within the upper digestive tract, allowing the detection of abnormalities.

**Figure 3:**
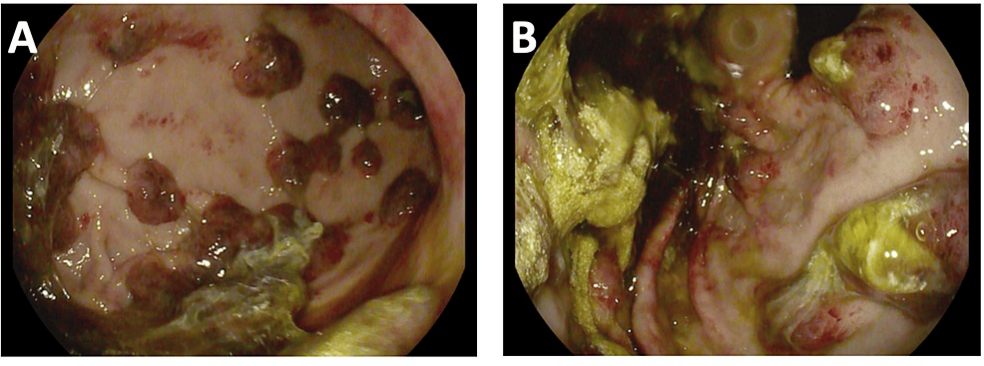
Endoscopic image of GI-KS in (a) the body of the stomach and (b) the stomach fundus GI-KS: gastrointestinal Kaposi sarcoma

Multiple lesions were observed, exceeding 100 in number, with a raised, polypoid appearance and purplish color, ranging from 5mm to 10mm in size. These lesions were found invading the gastric fundus, body, and antrum, consistent with GI-KS (Figure 3). Despite these lesions, no sign of active bleeding was observed during the procedure, and thus, no endoscopic therapy was required. Additionally, the duodenum appeared free of lesions. The procedure concluded without early or late complications, such as perforation, aspiration, or post-procedure bleeding.

Throughout the procedure, the endoscopist meticulously maneuvered the Pentax endoscope, adjusting its direction and depth to explore the entire length of the upper gastrointestinal tract. Biopsies were obtained using specialized tools passed through the working channel of the equipment, allowing histological examination of suspicious lesions. Subsequent histological analysis of the biopsied tissues confirmed the presence of KS, validating the initial clinical suspicion. 

After completion of the procedure, the patient was transferred back to the care of the attending physician with stable signs. Over the following weeks, the patient’s health progressively deteriorated, with hemoglobin levels dropping to 4.9 g/dl and leukocyte count rising to 13.7, with 91% neutrophils. Additionally, the lesion on the right leg became infected with Escherichia coli. Despite these complications, there were no further reports of gastrointestinal symptoms. The patient remained under the care of the attending physician in the respective department, with a probable diagnosis of immune reconstitution inflammatory syndrome. 

## Discussion

In individuals with AIDS-related KS (AIDS-KS), extracutaneous lesions are frequently observed in the GI tract, with a prevalence exceeding 50%. These lesions are distributed throughout the GI tract, with approximately 12-24% affecting the upper tract, 8-12% the lower tract, and 15-20% affecting both [2,7]. Given that merely 20% of these patients showcase the signs and symptoms above, the significance of prompt diagnosis lies in facilitating an effective decision-making process [8].

KS diagnosis can occur at any phase of HIV infection, but it is frequently associated with advanced immune deficiency, particularly in individuals with high HIV viral loads. When examined using endoscopy, GI-KS can present a spectrum of visual appearances, ranging from erythematous lesions to maculopapular or polypoid formations [9]. While most cases of GI-KS can be readily discerned during endoscopic examination, specific scenarios may present challenges as the lesions can mimic typical benign conditions such as peptic ulcers or granulation tissue, along with malignant neoplasms like gastrointestinal stromal tumor (GIST), spindle cell melanoma, and angiosarcoma [10,11].

Due to these factors, obtaining biopsy specimens for confirmatory testing through histopathology and immunohistochemistry is imperative. However, obtaining a conclusive endoscopic biopsy diagnosis is limited to 15-23% of cases, primarily to the tumor’s submucosal growth pattern. When there is no mucosal invasion, biopsies taken during endoscopy may only sample superficial tissue layers, posing challenges in identifying crucial histopathological features [12]. This approach is supported by findings from Silva et al., who reported similar endoscopic findings in a case report of GI-KS [13], as well as by Altamimi et al., who described a patient with a fleshy, large angioproliferative mass in the lesser curvature of the stomach [14]. However, they presented patients with few lesions. 

On the other hand, Jiménez et al. presented a case series of four patients in which one had multiple lesions covering the stomach; likewise, their patient did not describe any gastrointestinal symptoms, but at the endoscopy, multiple lesions were evident [15]. Aliaga-Ramos et al. also presented a case series of 13 patients, with just one presenting with digestive bleeding. Furthermore, 12 out of 13 patients presented more than three lesions characteristic of KS [16]. However, in our case, there were more evident lesions during the upper endoscopy, rendering it rare in clinical practice. This amount poses a more significant challenge in terms of management and suggests a potentially more aggressive disease course. 

## Conclusions

In summary, the multifaceted nature of KS, mainly its association with HHV-8, underscores the complexity of its clinical presentation and management. While AIDS-related KS represents the most aggressive form, gastrointestinal lesions, prevalent in AIDS patients, pose diagnostic challenges due to variable symptomatology. 

Timely accurate biopsy confirmation, supplemented by techniques like endoscopic ultrasound-guided biopsy when necessary, is imperative for guiding effective therapeutic strategies and optimizing patient outcomes in this diverse disease spectrum. 
